# Supplementary material for: Cyclodehydrogenation of molecular nanographene precursors catalyzed by atomic hydrogen
Source: Nat Commun. 2025 Jan 15;16:691. doi: 10.1038/s41467-024-54774-1 (PMC11735845; doi:10.1038/s41467-024-54774-1)
Supplement: Supplementary file 1 — Supplementary Information [file 41467_2024_54774_MOESM1_ESM.pdf]

Supplementary Information for

Cyclodehydrogenation of molecular nanographene precursors catalyzed by atomic hydrogen

Rafal Zuzak, Pawel Dabczynski, Jesús Castro-Esteban, José Ignacio Martínez, Mads Engelund, Dolores Pérez, Diego Peña, and Szymon Godlewski

Corresponding author: szymon.godlewski@uj.edu.pl, diego.pena@usc.es

This file discusses the following topics related to the main manuscript:

Supplementary Note 1: Hydrogen cracker

Supplementary Note 2: ToF-SIMS measurements

Supplementary Note 3: Additional data for 7-AGNRs and nanographenes **2** and **3** on Au(111)

Supplementary Note 4: Atomic hydrogen catalyzed cyclodehydrogenation reaction pathway

Supplementary Note 5: High coverage STM images of nanographenes **3** on TiO_2(110)-(1×1) and TiO_2(011)-(2×1)

Supplementary Note 6: Nanographenes **3** on NaCl/Cu(111)

Supplementary Note 7: Nanoflakes **3** on bulk NaCl

Supplementary References

Supp. Note 1: Hydrogen cracker

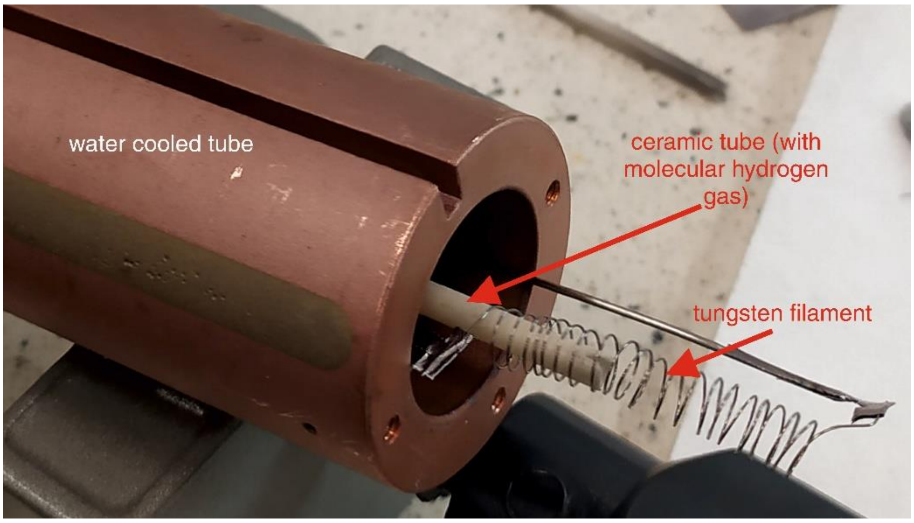

Suppl. Fig. 1. Hydrogen cracker. Detailed image of the home-built hydrogen cracker used in the experiments.

Supp. Note 2: ToF-SIMS measurements

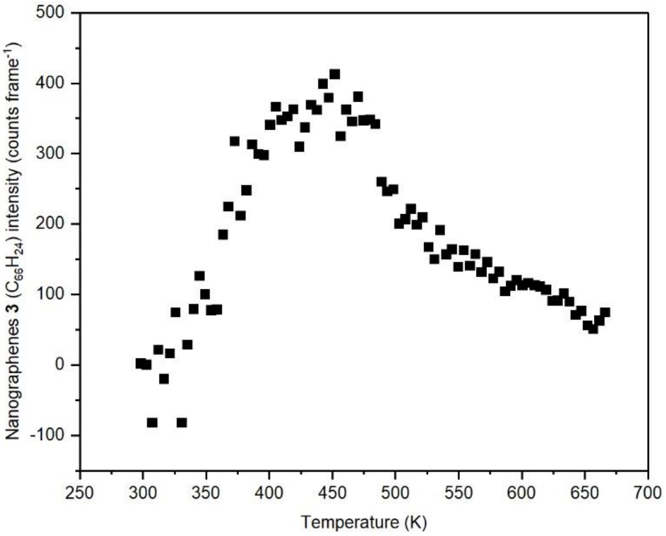

Suppl. Fig. 2. Nanographenes 3 generated during ToF-SIMS measurements on SiO_2. Intensity of $C_{66}H_{24}$ nanographene **3** flakes synthesized by atomic H generated during ToF-SIMS measurements as a function of temperature with subtracted influence of SIMS induced dehydrogenation – defined in equation (1) – methods section. Source data are provided as a Source Data file.

Supp. Note 3: Additional data for 7-AGNRs and nanographenes 2 and 3 on Au(111)

Suppl. Fig. 3a,b,c shows STM images with 7-AGNRs obtained through planarization by the application of atomic hydrogen. In these experiments the sample temperature was stabilized at 160, 180 and 220 °C during exposure to atomic hydrogen source. The experiments were performed in order to optimize the parameters for efficient planarization. As clearly discernible in Suppl. Fig. 3a,b,c the efficiency of cyclodehydrogenation was changed with the temperature. In all three images the not fully planarized 7-AGNRs could be identified by bright lobes corresponding to the alternate orientation of neighboring anthracene units. While there are only a few examples recorded for 7-AGNRs planarized at 220 °C (Suppl. Fig. 3c,

below 1% of not created C-C bonds) one could easily notice numerous not fully planarized units at 180 °C in Suppl. Fig. 3b and even more at 160 °C visualized in Suppl. Fig. 3a. Additionally we have also performed experiments with precursors **6** to analyze the influence of atomic H on the formation of nanographenes **3** (Suppl. Fig. 3e). Suppl. Fig. 3f,g shows perfect nanoflakes **3** generated at 220 °C and exposed to atomic hydrogen flux. Although transformation **6** → **3** could be initiated at 220 °C without atomic hydrogen images Suppl. Fig. 3f,g prove that atomic hydrogen does not lead to formation of methylene units leaving armchair- type edges in perfect shaping. For the sample containing precursors **6** and exposed to atomic hydrogen at 160 °C we have not achieved full planarization (precursors **6** are also not fully planarized into **3** at 160 °C without atomic hydrogen) indicating that the atomic hydrogen does not lead to cyclodehydrogenation temperature lowering. Finally we demonstrate that nanographenes **2** (Suppl. Fig. 3j) cannot be formed without atomic hydrogen by annealing at 220 °C (Suppl. Fig. 3k, self-assembly of precursors **5** into dimers), while are fully planarized at 220 °C with the application of atomic hydrogen (Suppl. Fig. 3l).

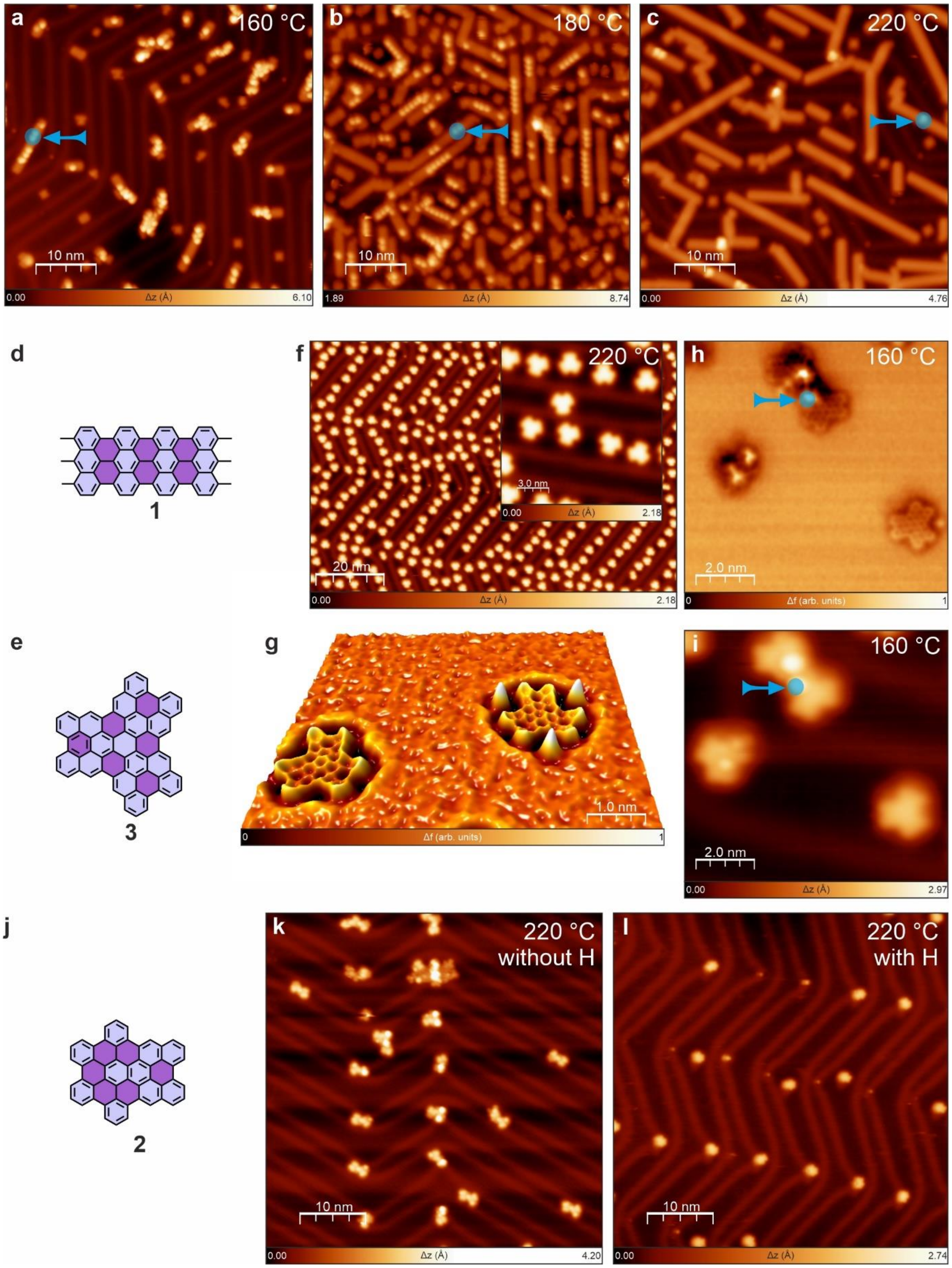

Suppl. Fig. 3. Cyclodehydrogenation on Au(111) with atomic hydrogen. STM images (**a**, **b** and **c)** show planarization of DBBA-based polymers with atomic hydrogen at 160, 200 and 220 °C, respectively. **d** Schematic image of 7-AGNR. **e** Scheme of nanographene **3**. STM (**f**) and nc-AFM (**g**) images of nanographenes **3** obtained on Au(111) by thermally initiated cyclodehydrogenation. The sample was exposed to atomic hydrogen source during annealing with no signs of influence on the reaction and final products. STM (**h**) and nc-AFM (**i**) images

of the very same fully and not-fully planarized precursors **6**. The sample was exposed to atomic hydrogen at 160 °C. **j** Schematic view of nanoflake **2**. Precursors **5** and nanoflakes **2** recorded in STM images on Au(111) after annealing at 220 °C without (**k**) and with (**l**) atomic hydrogen. Light violet marks hexagonal rings present in molecular precursors, whereas dark violet in **1**, **2**, **3** indicates rings generated through hydrogen catalyzed cyclodehydrogenation. Light blue arrows and circles indicate not fully planarized units. Bias voltage: -1 V, tunneling current: 15 pA (**k**, **l**), 20 pA (**a**, **b**), 25 pA (**i**), 50 pA (**c**, **f**). Source data are provided as a Source Data file.

The efficiency of the cyclodehydrogenation initiated by atomic H at 220 °C has been estimated as follows:

(i) from a large scale image (Fig. 2a in the main text) we have approximated the number of all possible new C-C bonds by measuring the lengths of GNRs; the estimated number of possible new C-C bonds is ≈ 8000

(ii) we have counted bright features located on the GNRs corresponding to non-fully planarized, unfinished units of GNRs; we could assume that each bright lobe corresponds to 1 (one bond on one side of the anthracene core is missing) or 2 (bonds on both sides of the anthracene unit are missing) missing C-C bonds

(iii) for rough estimation we assume that each bright lobe corresponds to 2 unformed bonds

(iv) the total number of bright lobes in Fig. 2a reaches 15

(v) the fraction of missing bonds is approximated by ≈ 30/8000=0.375 %<1 %

Supp. Note 4: Atomic hydrogen catalyzed cyclodehydrogenation reaction pathway

The experimental evidence demonstrates that the cyclodehydrogenative planarization reaction proceeds due to the catalytic atomic hydrogen action regardless of the substrate type. On this basis, in order to propose a plausible reaction mechanism, we have carried out a large battery of gas-phase Density Functional Theory (DFT)-based calculations to structurally and energetically characterize the different steps involved in the reaction path, as well as the transition states and associated energy barriers for each sub-reaction. As a proof-of-concept, we have considered pentahelicene (**9**) as a model compound to perform the gas-phase calculations, representing the molecular fjord region involved in the sequential cyclodehydrogenative planarization of compound **6**. For that purpose, we propose a reaction mechanism based in (see Suppl. Fig. 4): (i) the atomic H addition to the peripheral C atom that forms a π-radical in the neighboring peripheral C atom and activates the molecule towards (ii) a cyclization by a C-C coupling into a six-membered ring, (iii) followed by three sequential Eley-Rideal hydrogen abstraction reactions associated with the formation of three gas-phase H_2 molecules. The whole pathway leads to the final full planarization of the initial pentahelicene (**9**).

In a first step, we have adopted the DFT-based M06-2X/6-311++G(d,p) level of theory [1-3], as implemented in the atomistic simulation package Gaussian16 [4], to compute geometry optimizations and vibrational frequency analysis for all the structures shown in Suppl. Fig. 4. M06-2X method with large basis sets has demonstrated to provide accurate optimized structures and an excellent electron correlation treatment for the case of PAHs [5]. Resulting optimized geometries of these calculations are shown in Suppl. Fig. 4a, with **9** being the pristine pentahelicene, **10** the pentahelicene with a hydrogenated *sp*^{3} C atom, **11** the intermediate structure where the induced cyclization has formed an additional six-membered ring, **12** and **13** the intermediates formed by sequentiall losses of H atoms in *sp*^{3} C atoms, and **14** the final planar cyclized structure.

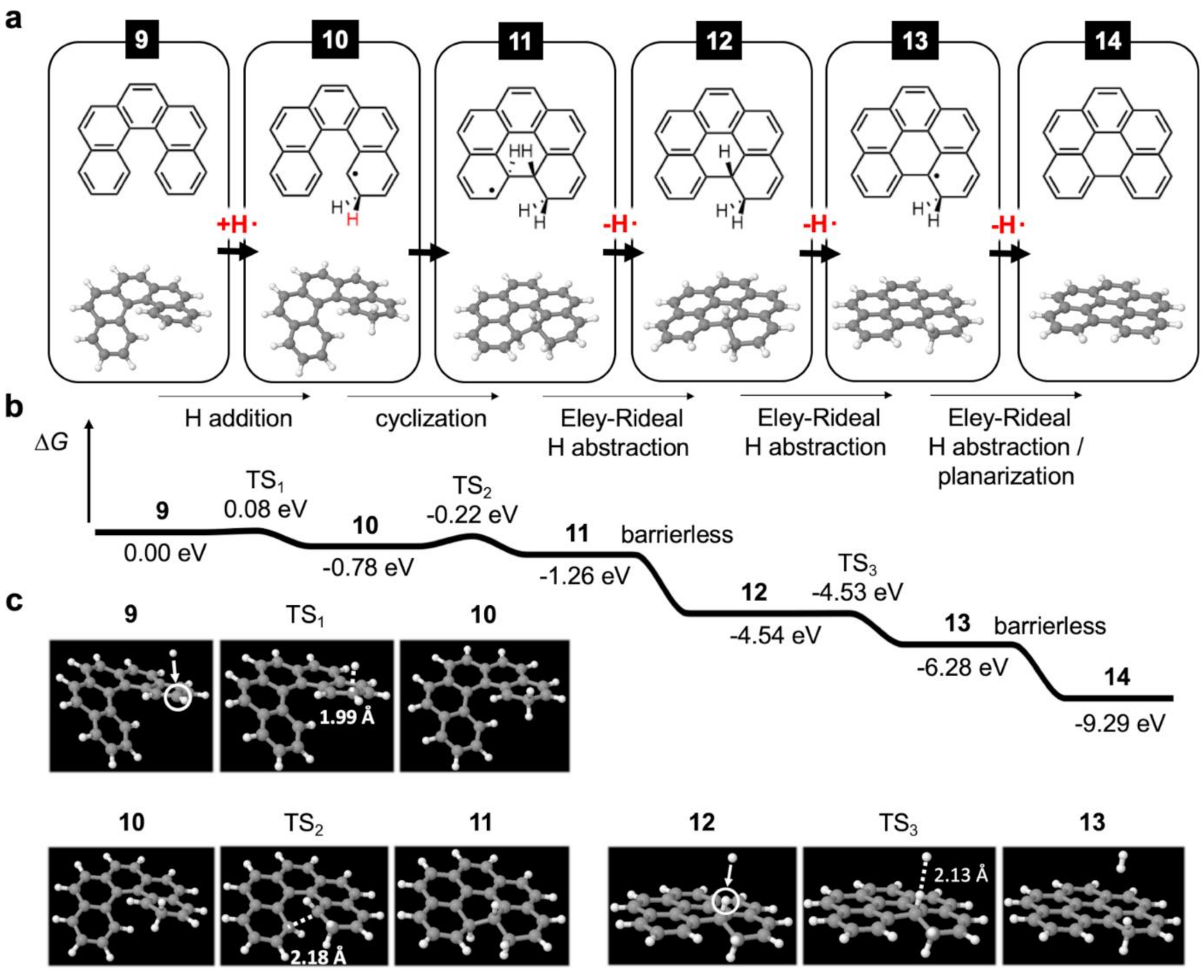

Suppl. Fig. 4. Mechanistic proposal for the atomic hydrogen induced planarization of the model compound. Proposal for the planarization of pentahelicene (**9**) to form benzo[*ghi*]perylene (**14**). **a** (top panel) Schematic structures of the precursor **9**, intermediates **10**-**13**, and planar product **14**, and (bottom panel) optimized ground-state molecular models corresponding to each reaction step. **b** Gibbs free-energy profile (in eV) by including entropic effects at 300 K. **c** Pictorial representation of the initial, transition and final states for the sub-reactions **9→10**, **10→11** and **12→13**, for which a non-null energy barriers have been found.

Once the initial, intermediate and final structures have been established, we have adopted the Climbing-Image Nudged Elastic Band (CI-NEB) formalism [6-8], as implemented in the plane-wave simulation package QUANTUM ESPRESSO [9], to compute the minimum energy paths (MEPs) and transition state energy barriers for all the sub-reactions involved in the cyclodehydrogenative planarization reaction mechanism shown in Suppl. Figure 4. These calculations have been performed within a cubical cell of 15×15×15 Å^3, sufficient to avoid residual interactions between neighboring computation cells, incorporating the GGA-PBESol functional [10] to address electronic exchange-correlation effects. The Grimme DFT-D3 semi-empirical efficient van der Waals correction [11] was applied to include dispersion forces and energies into conventional DFT functionals. Fully relativistic Kresse-Joubert projector-augmented wave (PAW) pseudopotentials [12] have been used to model the ion-electron interactions within atomic species. One-electron wave-functions were expanded in a basis of plane-waves with kinetic energy cutoffs of 36 and 280 Ry for kinetic energy and electronic density, respectively, ensuring sufficient accuracy for total energy and electronic density convergence. Within the CI-NEB approach the initial, final, and a sufficient number of intermediate image-states (20 in the present case) for each sub-reaction were free to fully

relax for the different systems. It is important to remark that, out of necessity, we use the approximation of a rigid barrier model, which should be acceptable up to temperatures of about 30% the melting temperatures of the systems.

On the basis of the results of these MEP and transition state energy barrier calculations, in conjunction with the Gibbs free energy of each structure, we can construct the full Gibbs free-energy profile shown in Figure S4b. The addition of a H atom to the peripheral C atom in **9→10** proceeds with a net energy gain of 0.78 eV, yielding a low energy barrier of 0.08 eV (see Suppl. Fig. 4b,c). This result is fully consistent with the previously reported values with similar net energy gains and barriers for the case of coronene [13-14]. The extra hydrogen added to the C atom activates the neighboring peripheral C atom by forming a π-radical and inducing the cyclization reaction **10→11** with a net energy gain of 0.47 eV and an energy barrier of 0.56 eV (see Suppl. Fig. 4b,c), which, according to the Boltzmann statistics, may be overcome in typical times of around 10^{-4} s. At this point, from the intermediate **11**, an H• + H• → H_2(g) associative mechanism between one H atom in one of the sp^3 C atoms and a H atom of the peripheral methylene group (-CH_2-) to release a H_2 gas-phase molecule is not feasible, since this mechanism yields an energy barrier > 3.5 eV. For that reason, given the availability of environmental atomic H coming from the H-cracker, we propose, from **11** to **14**, three Eley-Rideal H abstraction reactions [13-15] to sequentially remove the "extra" H atoms attached to sp^3 C atoms in the intermediate structures by the formation of subsequent H_2 molecules to finally achieve the full planarization of the molecule. **11→12** and **12→13** proposed Eley-Rideal H abstractions (by the formation of associated H_2 molecules) occur with no barrier, and with a small barrier of 0.01 eV, respectively, and with large net energy gains of 3.29 and 1.74 eV, respectively. The small barrier obtained for the **12→13** reaction (as compared with the absence of barrier for the **11→12**) has its origin in that the product **13** of this step is an electronic open-shell system, unlike the closed-shell **12**. This barrierless mechanism is also predicted for the proposed final **13→14** Eley-Rideal step - to remove the peripheral extra H in the formation of a H_2 molecule, achieving the full planarization of the molecule (also an electronic closed-shell) – with a net energy gain of 3.00 eV, which excellently agrees with previously reported data [13, 15].

In conclusion, these set of calculations permits to propose an atomistic viable mechanism for the cyclodehydrogenative planarization evidenced by the experiment, where the limiting reaction step is the C-C bond formation step **10→11**, with a moderate energy barrier of 0.56 eV. We not here that in the limit of the most favorable scenario, the hydrogenation of the two peripheral C atoms at both sides of the cycling C-C coupling atoms could substantially reduce this barrier (or remove it completely). However, such process is expected to be characterized by an extremely low occurrence probability.

Supp. Note 5: High coverage STM images of nanographenes 3 on TiO_2(110)-(1×1) and TiO_2(011)-(2×1)

In order to demonstrate the versatility and effectiveness of the planarization initiated by atomic hydrogen we have attempted generation of a closed layer of nanographenes **3** on two different faces of rutile titania, i.e., TiO_2(110)-(1×1) and TiO_2(011)-(2×1). Formation of flat lying nanoflakes **3** on both faces is visualized by empty-state high resolution images in Suppl. Fig. 5a,b with features clearly reflecting the shape of **3**.

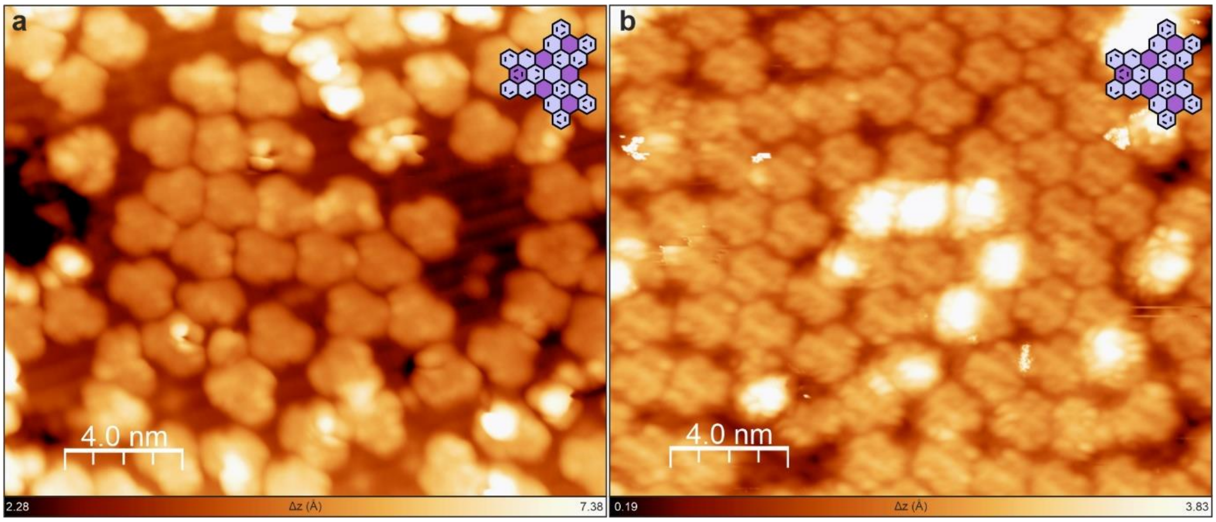

Suppl. Fig. 5. Nanographenes 3 on TiO_2. Empty-state high resolution STM images of **3** fabricated on (**a**) TiO_2(110)-(1×1) and (**b**) TiO_2(011)-(2×1). In both cases the STM parameters are adjusted for non-resonant tunneling through **3** thus the images resemble the geometric shape of **3**. Light violet marks hexagonal rings present in molecular precursor **6**, whereas dark violet in **3** indicates rings generated through hydrogen catalyzed cyclodehydrogenation. Bias voltage: +1.5 V, tunneling current: 20 pA (**a**), 25 pA (**b**). Source data are provided as a Source Data file.

Additional comparison of STM images acquired for precursors **6** on TiO_2(011)-(2x1), as well as nanographenes **3** on TiO_2(110)-(1x1) and TiO_2(011)-(2x1) is shown in Suppl. Fig. 6.

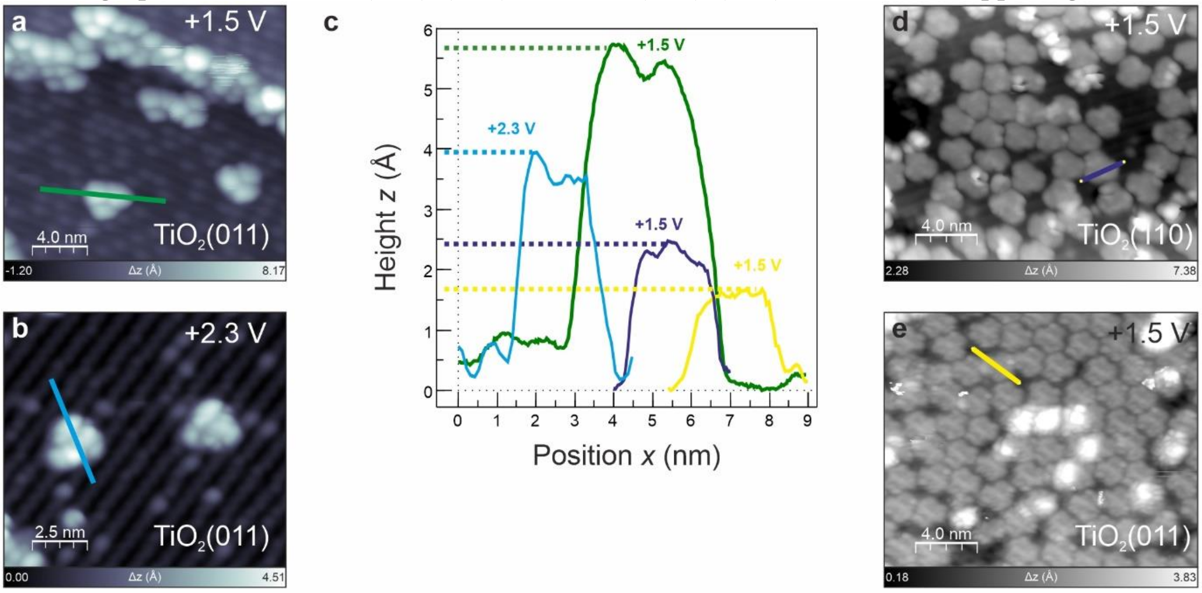

Suppl. Fig. 6. Precursors 6 and nanographenes 3 on TiO_2. STM images of precursors **6** on TiO_2(011)-(2×1) (**a**) and nanographenes **3** on TiO_2(011)-(2×1) (**b**, **e**) and TiO_2(110)-(1×1) (**d**) together with cross-section profiles (**c**) acquired along colored lines in (**a**, **b**, **d**, **e**): profile green across precursor **6** on TiO_2(011), profile blue and violet over nanographene **3** on TiO_2(011) and (110), respectively, profile yellow over nanographene **3** on TiO_2(011). Bias voltage: +1.5 V (**a**, **d**, **e**), +2.3 V (**c**), tunneling current: 10 pA (**a**), 15 pA (**b**), 20 pA (**d**), 25 pA (**e**). Source data are provided as a Source Data file.

Supp. Note 6: Nanographenes 3 on NaCl/Cu(111)

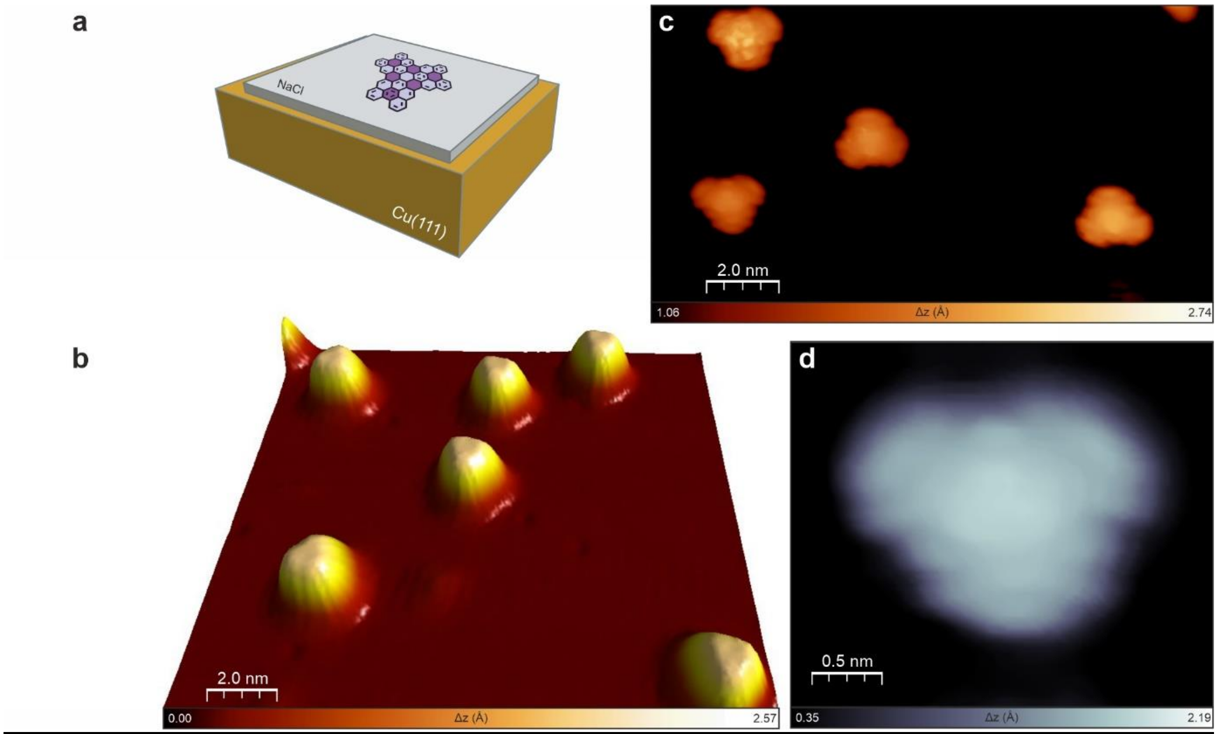

Suppl. Fig. 7. Nanographenes 3 on insulating film. Cyclodehydrogenation on a thin insulating NaCl layer on Cu(111). **a** Schematic view of NaCl/Cu(111) with nanographenes **3** on top. Empty (**b**) and filled state (**c**, **d**) images of nanographenes **3** on NaCl/Cu(111). Tunneling current: (**c**, **d**) 10 pA, (**b**) 30 pA, bias voltage: -1.5 V (**c**, **d**), +2.3 V (**b**). Source data are provided as a Source Data file.

The initiation of cylodehydrogenation by atomic hydrogen on semiconducting TiO_2 surfaces encouraged us to attempt the trial to generate nanoflakes **3** on a thin insulating layer placed on a metallic substrate (Suppl. Fig. 7a) that would allow identification of the reaction product by means of STM. In order to achieve the goal we have prepared the thin layer of NaCl on Cu(111) and subsequently deposited molecular precursors **3** on top. Further the system was exposed to atomic hydrogen flux at 220 °C. Suppl. Fig. 7 shows STM images of nanoflakes **3** indicating on the successful transformation **6** → **3**. Figure S7b shows a 3D empty state STM topography with clearly discernible triangular features. High resolution images are visualized in Suppl. Fig. 7c,d.

Supp. Note 7: Nanoflakes 3 on bulk NaCl

In order to verify ability for the synthesis of nanographenes **3** on bulk insulator we have deposited precursors **6** onto bulk NaCl in UHV. Due to inability to apply STM we have chosen ToF-SIMS as a technique to monitor the transformation from **6** into planar nanographene **3**. To avoid contamination of the hydrophilic NaCl sample (e.g. by water), which would influence ToF-SIMS measurements (observed in test experiments) we have applied the vacuum vessel to transport the samples from the preparation chamber of the LT-STM/AFM system into the ToF-SIMS. The nc-AFM image of the NaCl(001) clean surface (without precursors, after atomic hydrogen treatment, as a reference) is shown in Suppl. Fig. 8. Due to the expected weak interaction of precursors **6** (and **3**) with NaCl and low desorption temperature we have performed the atomic hydrogen treatment at lower temperature, e.g. 180 °C to avoid desorption of molecules from the sample.

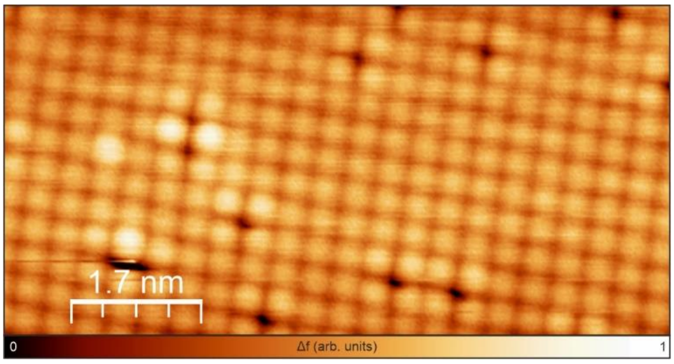

Suppl. Fig. 8. NaCl exposed to atomic hydrogen. Nc-AFM image of NaCl(001) bulk crystal recorded after atomic hydrogen treatment. Source data are provided as a Source Data file.

The static ToF-SIMS data obtained for the substrate with precursors **6** are shown in Suppl. Fig. 9 displaying the normalized intensity of both $C_{66}H_{36}$ and $C_{66}H_{24}$ during annealing in the ToF-SIMS setup and indicating that molecules desorb from the surface within a similar temperature range and are undetectable after annealing at 185 °C. Therefore we expect the desorption to be the limiting factor for the transformation of **6** into **3**.

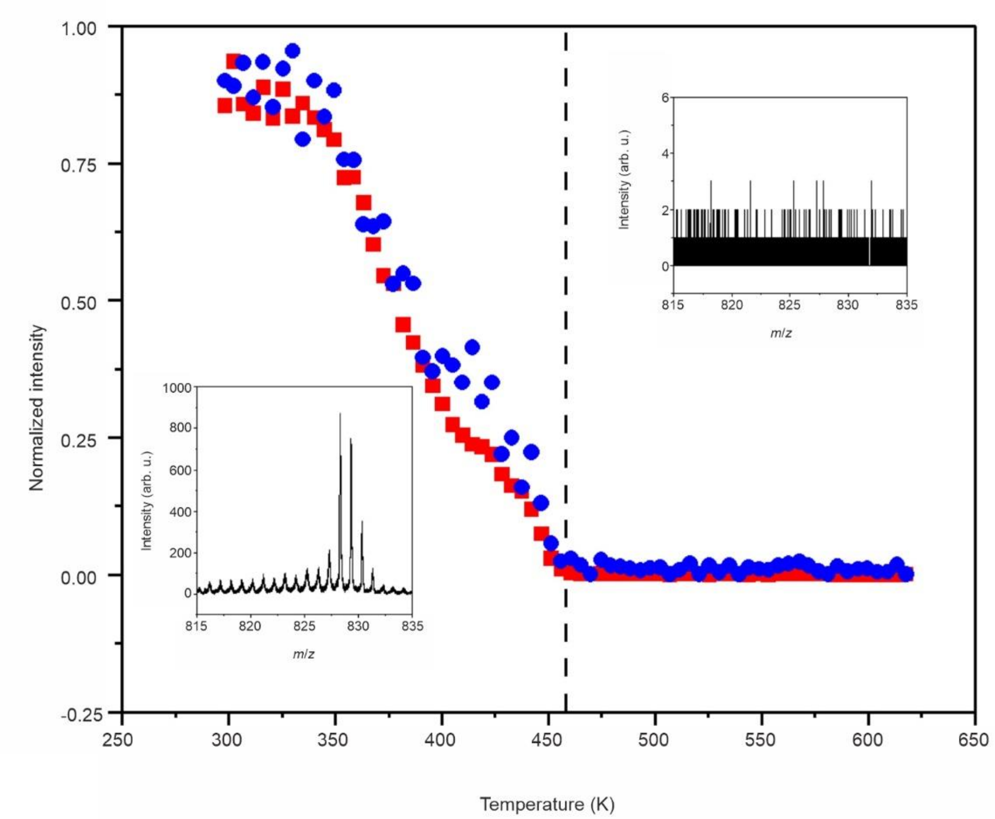

Suppl. Fig. 9. Precursors 6 and nanographenes 3 on bulk NaCl. Static ToF-SIMS $C_{66}H_{36}$ (mass 828.286 g mol^{-1} – red) and $C_{66}H_{24}$ (mass 816.134 g mol^{-1} – blue) signals as a function of temperature obtained for the NaCl sample containing precursors **6**. Insets show mass spectra integrated: from room temperature 25 °C up to 185 °C (left bottom corner), from 185 °C to 345 °C (right top corner). Data point – to – point normalized to the reference experiment conducted with the same primary ion dose density at room temperature. Source data are provided as a Source Data file.

The ToF-SIMS spectra acquired for samples containing only precursors **6** ($C_{66}H_{36}$) and obtained after atomic hydrogen treatment of **6** ($C_{66}H_{36}$) on NaCl at 180 °C are displayed in

Suppl. Fig. 10 in blue and brown, respectively.The spectra show presence of precursors **6** on the sample before hydrogen treatment and demonstrate recording a mixture of both precursors **6** and nanoflakes **3** after atomic hydrogen treatment at 180 °C, although the efficiency is limited due to the desorption.

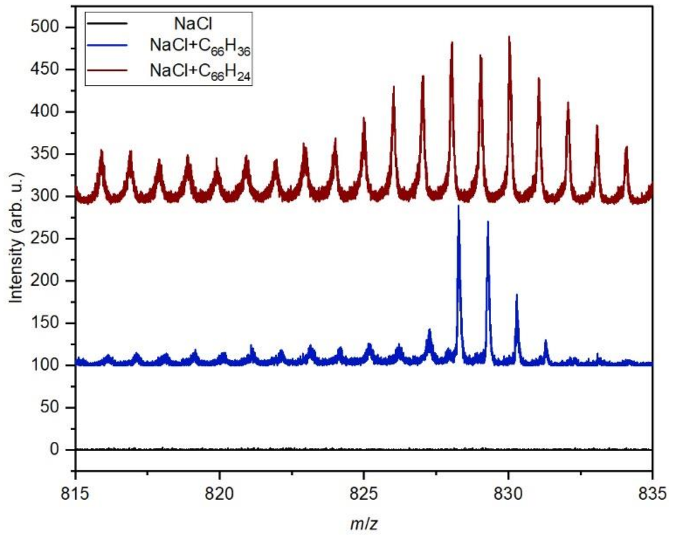

Suppl. Fig. 10. Nanographenes 3 on bulk NaCl. ToF-SIMS spectra acquired for nanoflake **3** ($C_{66}H_{24}$) synthesis on bulk NaCl. $C_{66}H_{24}$ and $C_{66}H_{36}$ mass regions are shown; black – reference NaCl, blue – spectra acquired for the sample with deposited precursors **6** ($C_{66}H_{36}$), red – sample with precursors **6** annealed at 180 °C with atomic hydrogen, the series of peaks starting at mass 816 g mol^{-1} show presence of nanographenes **3** on the sample. All NaCl samples were transported from the preparation chamber into the ToF-SIMS setup with vacuum vessel. Source data are provided as a Source Data file.

Supplementary References

1. Zhao, Y., Truhlar, D.G. The M06 suite of density functionals for main group thermochemistry, thermochemical kinetics, noncovalent interactions, excited states, and transition elements: two new functionals and systematic testing of four M06-class functionals and 12 other functionals, *Theor. Chem. Acc.* **120**, 215-241 (2008).

2. Krishnan, R., Binkley, J.S., Seeger, R., Pople, J.A. Self-consistent molecular orbital methods. A basis set for correlated wave functions, *J. Chem. Phys.* **72**, 650-654 (1980).

3. McLean, A., Chandler, G. Contracted Gaussian basis sets for molecular calculations. I. Second row atoms, Z= 11–18, *J. Chem. Phys.* **72**, 5639-5648 (1980).

4. Frisch, M., Trucks, G., Schlegel, H., Scuseria, G., Robb, M., Cheeseman, J., Scalmani, G., Barone, V., Petersson, G., Nakatsuji, H. Gaussian 16, Gaussian, Inc, Wallingford, CT (2016).

5. Li, Y., Wang, T., Yalamanchi, K.K., Kukkadapu, G., Sarathy, S.M. Accurate thermochemistry prediction of extensive polycyclic aromatic hydrocarbons (PAHs) and relevant radicals, *Combustion and Flame* **242**, 112159 (2022).

6. Henkelman, G., Jónsson, H. Improved Tangent Estimate in the Nudged Elastic Band Method for Finding Minimum Energy Paths and Saddle Points. *J. Chem. Phys.* **113**, 9978–9985 (2000).

7. Classical and Quantum Dynamics in Condensed Phase Simulations; Berne, B. J., Ciccotti, G., Coker, D. F., Eds.; WORLD SCIENTIFIC, 1998.

8. Henkelman, G., Uberuaga, B.P., Jónsson, H. A Climbing Image Nudged Elastic Band Method for Finding Saddle Points and Minimum Energy Paths. *J. Chem. Phys.* **113**, 9901-9904 (2000).

9. Giannozzi, P., Baroni, S., Bonini, N., Calandra, M., Car, R., Cavazzoni, C., Ceresoli, D., Chiarotti, G.L., Cococcioni, M., Dabo, I. QUANTUM ESPRESSO: a modular and open-source software project for quantum simulations of materials. *J. Phys.: Cond. Matter.* **21**, 395502 (2009).
10. Perdew, J.P., Ruzsinszky, A., Csonka, G.I., Vydrov, O.A., Scuseria, G.E., Constantin, L.A., Zhou, X., Burke, K. *Phys. Rev. Lett.* **100**, 136406 (2008).
11. Grimme, S. Semiempirical GGA-type density functional constructed with a long-range dispersion correction. *J. Comput. Chem.* **27**, 1787-1799 (2006).
12. Kresse, G., Joubert, D. From Ultrasoft Pseudopotentials to the Projector Augmented-Wave Method. *Phys. Rev. B* **59**, 1758 (1999).
13. Rauls, E., Hornekaer, L. Catalyzed routes to molecular hydrogen formation and hydrogen addition reactions on neutral polycyclic aromatic hydrocarbons under interstellar conditions. *Astrophys. J.* **679**, 531-536 (2008).
14. Barrera, N.F., Fuentealba, P., Muñoz, F., Gómez, T., Cárdenas, C. Formation of H_2 on polycyclic aromatic hydrocarbons under conditions of the ISM: an abinitio molecular dynamics study, *Mon. Notices Royal Astron. Soc.* **524**, 3741-3748 (2023).
15. Ferullo, R.M., Zubieta, C.E., Belelli, P.G. Hydrogenated polycyclic aromatic hydrocarbons (HnPAHS) as catalysts for hydrogenation reactions in the interstellar medium: a quantum chemical model, *Phys. Chem. Chem. Phys.* **21**, 12012-12020 (2019).
